# Letter: the limitations of gene set-based predictive models: a critical assessment

**DOI:** 10.1186/s12967-025-06476-5

**Published:** 2025-05-08

**Authors:** Lijing Yang, Wenbo Zhao, Min Jiang, Xianqiang Liu

**Affiliations:** 1 The First Affiliated Hospital, and College of Clinical Medicine of Henan University of Science and Technology, Luoyang, 471000 China; 2 Medical School of Chinese PLA, Beijing, 100853 China; 3 Shandong First Medical University, Jinan, 250117 China

Predefined gene set-based predictive models are increasingly applied in the field of translational bioinformatics [1]. Many studies have developed models based on gene sets associated with specific biological processes, such as ferroptosis (728 genes) [2] and lactylation (374 genes) [3]. This approach has led to a surge in related research, with more than 1,000 such studies reported in 2024 alone. However, the biological specificity and validity of many gene set models have not been fully validated, raising concerns about their scientific and clinical value. To critically evaluate the robustness of such models, we analyzed three cancers with the highest mortality rates: lung, colorectal, and liver cancer [4], utilizing data from The Cancer Genome Atlas (TCGA). After performing univariate Cox regression for each cancer type, we identified genes significantly associated with patient survival, with 2,011 genes for colorectal cancer, 2,211 for lung cancer, and 6,324 for liver cancer. We then randomly selected gene sets of sizes ranging from 20 to 500 genes and repeated the process 100 times for each cancer. Nearly all randomly selected gene sets successfully stratified patients by survival outcome. Moreover, as the number of genes in the gene set increased, model performance significantly improved, with larger gene sets yielding higher accuracy, as reflected in higher AUC values, hazard ratios, and more significant p-values across the three cancer types (see Fig. 1). These results suggest that the success of many gene set-based models may stem more from statistical chance or model flexibility than from real biological mechanisms.

**Fig. 1:**
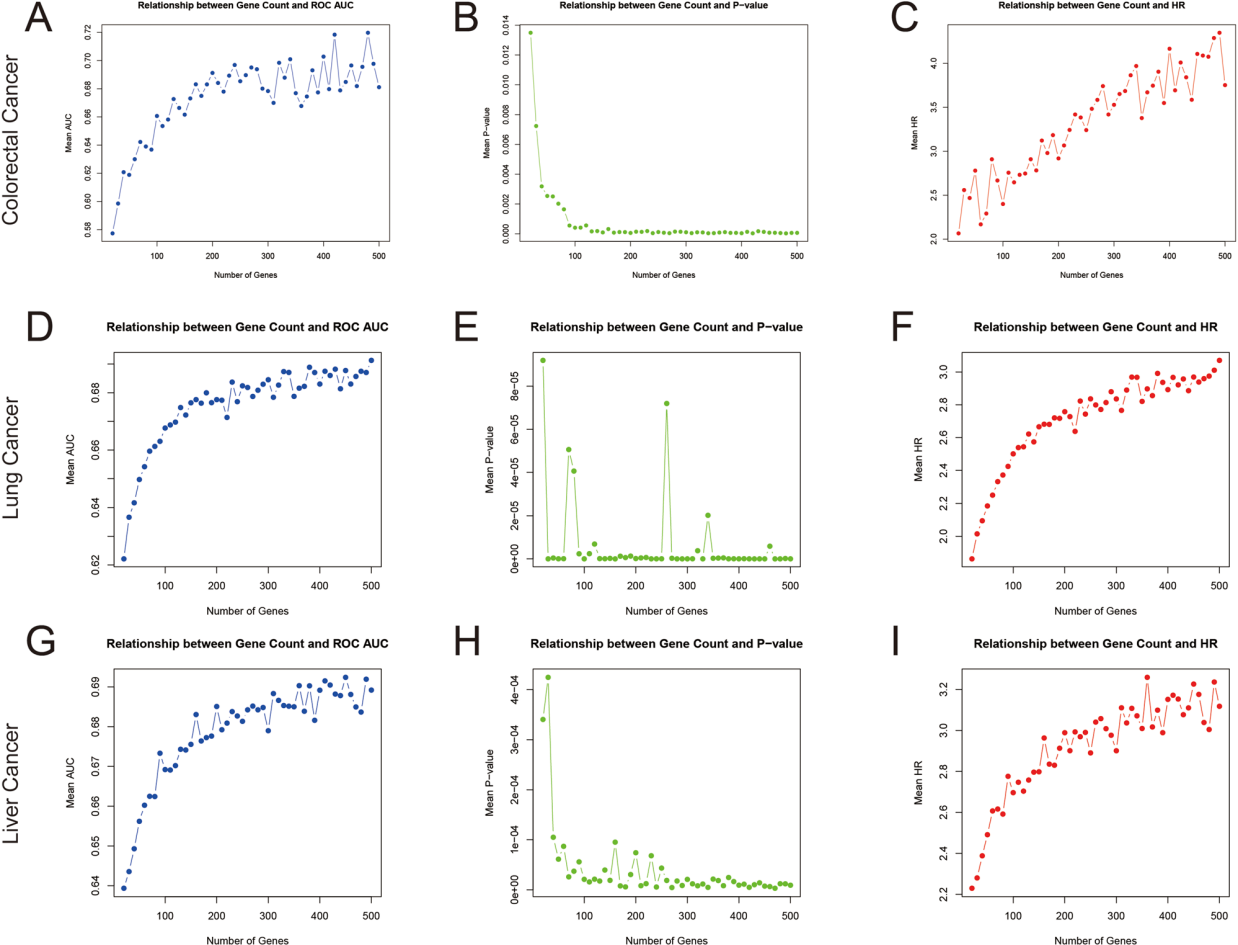
The relationship between gene set size and prognostic model performance across three cancer types. To examine the relationship between gene set size and prognostic model performance, correlation analysis was performed, with all analyses repeated 100 times. For colorectal cancer: panel (**A**) shows the relationship between AUC and gene set size, (**B**) shows the relationship between gene set size and log-rank p-value, and (**C**) shows the relationship between gene set size and hazard ratio (HR). Similarly, panels (**D**), (**E**), and (**F**) show the corresponding relationships for lung cancer, and panels (**G**), (**H**), and (**I**) show the corresponding relationships for liver cancer

Our analysis reveals that arbitrary gene sets can easily achieve significant survival stratification, highlighting a major issue with gene set models. While many studies include some experimental validation of their models, the biological specificity of these models is often insufficiently explored. Statistically significant models may not necessarily reflect the true disease mechanisms. In practice, predefined gene sets are often used to construct predictive models without rigorous functional validation, raising concerns that their performance may be driven by statistical artifacts, cohort-specific biases, or overfitting, rather than true disease pathology. This issue is not limited to prognostic models, but also affects classification models used for disease diagnosis, immunotherapy response prediction, and chemo-radiotherapy sensitivity assessment. Even if these models show statistical significance, they may lack real clinical relevance. Despite these shortcomings, gene set models can still serve as valuable exploratory tools in some contexts, such as generating hypotheses about disease-related pathways or guiding basic research.

Another significant limitation of current predictive modeling is the lack of normal (healthy) control data for comparison. In TCGA, most tumor datasets lack matched normal tissue samples. The absence of normal controls introduces systematic biases, making it difficult to differentiate disease-specific molecular signals from background noise, further reducing the biological relevance of computational models. To improve the reliability and interpretability of gene set-based predictions, future studies should aim to include diverse patient cohorts and ensure proper matching with normal control samples. Even if a gene set model performs well statistically, it must undergo comprehensive biological validation to be considered clinically meaningful. Many computational biomarkers that initially seemed promising have failed to translate into clinical practice due to the lack of mechanistic validation or functional assays. To avoid this pitfall, future research must validate predictive models across independent cohorts and confirm findings through laboratory experiments. For example, integrating multi-omics data (such as genomics, transcriptomics, proteomics, and metabolomics) will provide a more holistic understanding of disease biology, help identify real molecular interactions, reduce false positives, and refine disease subtype classification. Additionally, direct experimental validations, such as functional assays and single-cell transcriptomic analysis, can verify whether the gene sets in a model truly affect the disease biology. Moreover, stricter statistical methods, such as permutation testing, cross-validation, and independent dataset validation, should be applied to prevent overfitting and ensure the model’s predictive power can be generalized beyond the original cohort. The widespread application of gene set-based predictive models highlights the urgent need for more rigorous standards in biomarker discovery and validation. Researchers should prioritize biologically interpretable and mechanistically grounded findings over mere statistical significance. Journals and funding agencies also play a crucial role in enforcing higher requirements, such as ensuring computational models are supported by experimental or clinical validation. By moving beyond purely statistical associations and focusing on biological relevance, we can ensure that new predictive models truly advance disease biology and ultimately improve patient outcomes.

## Data Availability

Not applicable.
